# Acute gastrointestinal hemorrhage as the sole initial manifestation of gastric leiomyosarcoma: a case report highlighting the discrepancy between preoperative assessment and final pathology

**DOI:** 10.3389/fonc.2026.1876041

**Published:** 2026-06-24

**Authors:** Kaini Li, Yuan Zhang, Liangchen Li, Liwei Liu, Shangyumeng Zhao, Chuanjie Yang

**Affiliations:** 1 Department of Gastroenterology, The Second Hospital of Hebei Medical University, Hebei Key Laboratory of Gastroenterology, Hebei Institute of Gastroenterology, Hebei Clinical Research Center for Digestive Diseases, Shijiazhuang, Hebei, China; 2 Department of General Surgery, The Second Hospital of Hebei Medical University, Shijiazhuang, Hebei, China; 3 Department of preventive medicine, College of Public Health, Hebei Medical University, Shijiazhuang, Hebei, China

**Keywords:** gastric leiomyosarcoma, gastrointestinal hemorrhage, immunohistochemistry, Ki-67, mesenchymal tumor

## Abstract

Gastric leiomyosarcoma (LMS) is an exceedingly rare mesenchymal malignancy, and its presentation with acute upper gastrointestinal hemorrhage is poorly documented. We present a case of this tumor in a 78-year-old female, whose sole initial manifestation was acute upper gastrointestinal hemorrhage with severe anemia (hemoglobin 70 g/L). Gastroscopy showed a 1.2 × 1.8 cm ulcerated submucosal protrusion; contrast-enhanced computed tomography (CT) revealed a 2.0 cm moderately enhancing gastric wall nodule without regional lymphadenopathy or distant metastasis. Endoscopic biopsy demonstrated a spindle cell neoplasm negative for CD117 and DOG1 but positive for SMA and SMMS-1, with a Ki-67 index of 25%. Laparoscopic wedge resection yielded a 5.0 × 3.0 × 1.5 cm tumor. Final pathology confirmed LMS with an elevated Ki-67 of 50% and a mitotic count of 13/10 high-power fields (HPF). This case underscores that superficial biopsy and preoperative imaging may substantially underestimate the true infiltrative extent and proliferative activity of gastric LMS. Acute hemorrhage may herald this rare malignancy despite seemingly unremarkable endoscopic findings. Accurate diagnosis mandates immunohistochemical exclusion of gastrointestinal stromal tumor (GIST). Close postoperative surveillance is warranted.

## Introduction

Gastric LMS is a rare malignant mesenchymal neoplasm originating from the smooth muscle cells of the gastric wall. According to the 2020 WHO Classification of Soft Tissue and Bone Tumours, it is categorized under smooth muscle tumors, a group that includes benign leiomyomas and malignant leiomyosarcomas (1). Epidemiologically, gastric LMS accounts for 1% of all malignant gastric tumors and is vastly outnumbered by gastrointestinal stromal tumors (GISTs), which represent the predominant mesenchymal neoplasm at this site (2). The vast majority of gastric smooth muscle tumors are benign leiomyomas, whereas LMS are exceptionally infrequent, with only sporadic case reports and small retrospective case series published in the literature to date. Given this scarcity, the clinical characteristics, diagnostic challenges, and optimal management strategies for gastric LMS remain incompletely defined.

Due to its infiltrative growth pattern and origin from the muscularis propria or muscularis mucosae layer in various locations of the stomach, early-stage gastric LMS typically presents with an oligosymptomatic or asymptomatic clinical course (3). As the disease progresses, the most common manifestations include abdominal pain, melena, weight loss, and the presence of an abdominal mass—symptoms that are nonspecific and readily misattributed to benign gastric disorders or other more prevalent gastric malignancies (4). Notably, acute upper gastrointestinal hemorrhage as the sole initial manifestation of gastric LMS is exceedingly rare, with only sporadic cases documented in the English-language literature (5). Such atypical presentations pose substantial diagnostic and therapeutic challenges, particularly in the emergency setting. Furthermore, gastric LMS arising in elderly patients over 75 years of age with multiple comorbidities is infrequently encountered, and evidence regarding optimal perioperative management and long-term surveillance strategies for this vulnerable population remains limited.

We herein report a case of primary gastric LMS in a 78-year-old female with multiple comorbidities, who presented with acute upper gastrointestinal hemorrhage as the sole initial manifestation. This case illustrates an uncommon clinical presentation and highlights notable histopathologic findings—specifically, a marked discrepancy between endoscopic and surgical tumor dimensions and a substantial escalation in the Ki-67 proliferation index—that may inform diagnostic vigilance and postoperative surveillance in similar cases. This case report was prepared in accordance with the SCARE (Surgical CAse REport) guidelines, and a completed SCARE checklist is provided as a supplementary file.

## Case presentation

A 78-year-old female presented to the emergency department on February 11, 2026, with a 1-day history of intermittent hematemesis and melena, accompanied by dizziness and palpitations. She reported no preceding acid reflux, heartburn, abdominal pain, unintentional weight loss, anorexia, or other constitutional symptoms. Her medical history was notable for a 7-year history of hypertension, a 3-year history of type 2 diabetes mellitus, coronary artery disease, and a prior cerebral infarction with residual left upper limb numbness. Her chronic diseases were managed with oral medications, and she specifically denied regular use of antiplatelet or anticoagulant agents. She denied any family history of hereditary disorders or neoplastic syndromes. Her personal history was unremarkable for prior major surgery, trauma, blood transfusion, or infectious diseases such as hepatitis and tuberculosis. No food or drug allergies were reported. The patient was a farmer living in a rural area with her family. She had no history of smoking or alcohol consumption, and no known exposure to occupational or environmental carcinogens. Her hypertension and diabetes had been managed with oral medications for several years with generally stable blood pressure and glycemic control, although no recent quantitative data were available at the time of admission.

On physical examination, the patient was alert and oriented, with conjunctival pallor and an overall anemic appearance. Vital signs were: temperature 36.3°C, pulse 81 beats/min, respiratory rate 20 breaths/min, and blood pressure 139/65mmHg. No jaundice was observed. Breath sounds were clear bilaterally, without wheezes or crackles. Heart rhythm was regular, with no murmurs appreciated over the precordium. The abdomen was flat and soft, with no tenderness, rebound, or guarding. Liver and spleen were not palpable. Shifting dullness was negative, bowel sounds were normoactive, and there was no lower extremity edema.

Emergency laboratory tests obtained on admission revealed a hemoglobin concentration of 70 g/L, consistent with severe acute hemorrhagic anemia. Emergency gastroscopy revealed a 1.2 × 1.8 cm protruding lesion with apical fissure-like ulceration on the greater curvature of the lower gastric body (**Figure 1**). A duodenal bulb ulcer (S2 stage) was also noted but exhibited no stigmata of recent bleeding and was therefore excluded as the source of hemorrhage. Abdominopelvic contrast-enhanced CT revealed a 2.0 cm nodule with moderate enhancement on the gastric greater curvature (**Figure 2**), with no evidence of regional lymphadenopathy or distant metastasis.

**Figure 1:**
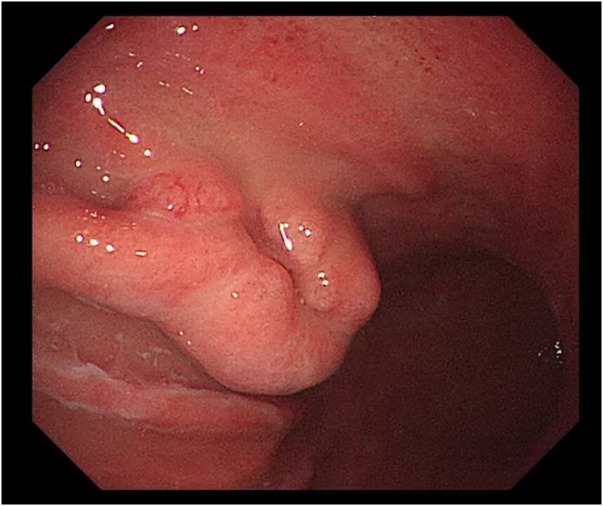
Endoscopic view of the gastric body showing a 1.2 × 1.8 cm protruding lesion with an apical fissure-like ulceration on the greater curvature.

**Figure 2:**
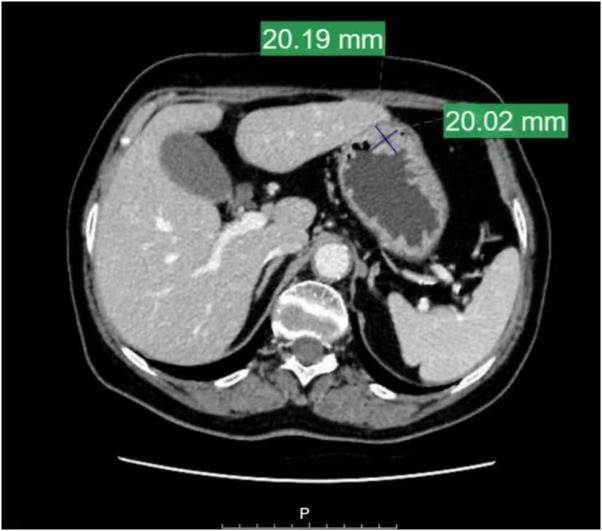
Contrast-enhanced CT (venous phase) showing a 2.0 cm nodule with moderate enhancement on the gastric greater curvature.

Given the endoscopic and imaging findings of a submucosal gastric lesion with apical ulceration and moderate contrast enhancement, the initial differential diagnosis was broad and included GIST, gastric leiomyoma, gastric schwannoma, and other less common mesenchymal neoplasms (6). Notably, the presence of mucosal ulceration with acute hemorrhage raised suspicion for a malignant process, as benign submucosal lesions such as leiomyomas rarely present with active bleeding. However, cross-sectional imaging alone could not reliably distinguish among these entities, necessitating histopathological and immunohistochemical evaluation (7).

Histopathologic examination of endoscopic biopsies revealed focal spindle cell proliferation in the lamina propria with cellular atypia and visible mitotic figures, set against a background of chronic active inflammation and granulation tissue. Immunohistochemical staining showed: CD117(−), DOG1(−), S-100(−), Desmin (focally positive), SMA(+), SMMS-1(+), and Ki-67 (approximately 25%). This immunoprofile confirmed a smooth muscle-derived neoplasm, favoring a diagnosis of LMS.

The definitive exclusion of GIST by immunohistochemistry (CD117/DOG1 double-negative) and the confirmation of smooth muscle differentiation (SMA and SMMS-1 positivity) narrowed the differential diagnosis to smooth muscle-derived tumors. The distinction between benign leiomyoma and malignant LMS, however, remained uncertain based on the limited superficial biopsy material alone, given the presence of cellular atypia and mitotic activity in the biopsy specimen. Complete histopathological evaluation of the fully resected tumor was therefore necessary to establish the final diagnosis, determine the FNCLCC grade, and guide postoperative risk stratification.

### Treatment

Given the patient’s acute hemorrhagic presentation, advanced age, and multiple comorbidities, laparoscopic wedge resection was selected as a minimally invasive approach to achieve both hemorrhage control and complete tumor removal while minimizing postoperative morbidity. Following multidisciplinary team (MDT) assessment and confirmation of surgical eligibility, the patient underwent elective laparoscopic wedge resection under general anesthesia on March 5, 2026, after full stabilization of her acute condition. Preoperatively, hemoglobin had improved to 98 g/L following transfusion and supportive care, with complete resolution of gastrointestinal hemorrhage. Intraoperative exploration revealed no ascites, peritoneal carcinomatosis, or distant metastases within the abdominal and pelvic cavities. Laparoscopic visualization identified a 2 × 2 × 1.5 cm firm nodule with an extraluminal growth pattern and intact serosa on the anterior gastric body. Notably, this corresponded to the same lesion visualized endoscopically as a 1.2 × 1.8 cm intraluminal protrusion with apical ulceration. The tumor was situated predominantly within the muscularis propria and exhibited both intraluminal and extraluminal extension, which accounted for the underestimation of its true dimensions by preoperative imaging and intraoperative inspection. The tumor was resected en bloc with negative margins, the gastric wall defect was closed using an endoscopic linear stapler, and a closed-suction drain was placed in the splenic fossa. No intraoperative blood transfusion was required, and the patient remained hemodynamically stable throughout the procedure with uneventful postoperative emergence.

Pathological examination of the resected specimen revealed a 5 × 3 × 1.5 cm solid spindle cell neoplasm with a mitotic count of 13 per 10 HPF (**Figure 3**). According to the FNCLCC grading system, the tumor was assigned a differentiation score of 2 (conventional LMS with marked atypia), a mitotic score of 2 (13/10 HPF), and a necrosis score of 0 (no tumor necrosis). The total score of 4 points corresponds to FNCLCC Grade 2. Supplementary immunohistochemistry confirmed the diagnosis of LMS, with the following profile: CD117(−), DOG1(−), SMA(+), SMMS-1(+), Desmin (focally positive), S-100(−), and Ki-67 (50%). All surgical margins were negative for tumor, confirming an R0 resection. No suspicious lymphadenopathy was noted intraoperatively, and no lymph nodes were harvested for pathological evaluation.

**Figure 3:**
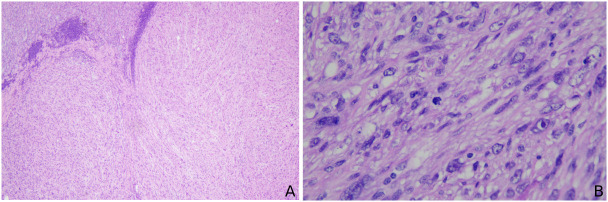
Histomorphological findings of the resected specimen (hematoxylin and eosin stain). **(A)** Low-power view (×100 magnification) showing intersecting fascicles of spindle cells with elongated nuclei, eosinophilic cytoplasm. **(B)** High-power view (×400 magnification) demonstrating a representative mitotic figure. The mitotic count was 13 per 10 high-power fields (HPF).

The postoperative course was uneventful with regard to surgical complications: no anastomotic leak, secondary hemorrhage, wound infection, or intestinal obstruction occurred. The patient was discharged on March 10, 2026, in stable condition. A MDT-directed surveillance strategy was established, with planned close follow-up including periodic gastroscopy and chest and abdominopelvic contrast-enhanced CT to monitor for local recurrence and distant metastasis. The timeline of clinical events and interventions for this patient is summarized in the figure (**Figure 4**). At the time of manuscript preparation, the patient was alive and attending regular follow-up visits as scheduled, with no clinical signs of recurrence reported.

**Figure 4:**
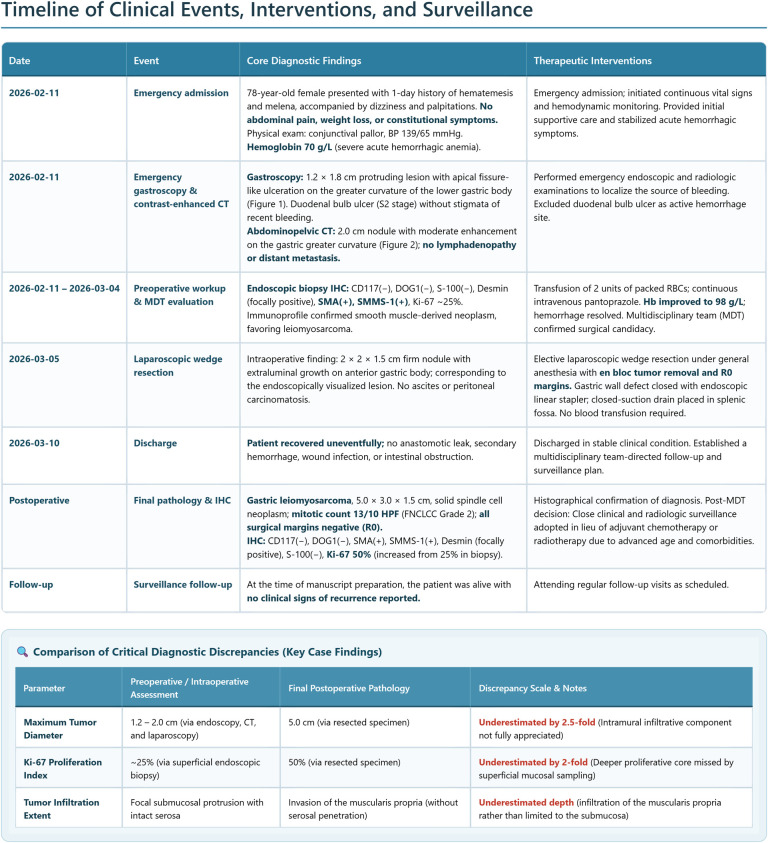
Chronological timeline of clinical events, diagnostic workup, therapeutic interventions, and follow-up for the patient with gastric leiomyosarcoma, including a comparison of critical diagnostic discrepancies. CT, computed tomography; FNCLCC, Federation Nationale des Centres de Lutte Contre le cancer; HFP, high-power fields; IHC, immunochemistry; MDT, multidisciplinary team; RDC, red blood cells.

## Discussion

Gastric LMS is an exceptionally rare mesenchymal malignancy, vastly outnumbered by GISTs. The reported median age at diagnosis is 59 years (8), and presentations in patients over 75 with significant comorbidities are infrequently documented. Clinical presentation is typically nonspecific, and the absence of early symptoms often leads to diagnosis at an advanced stage. The most striking feature of the present case is that acute upper gastrointestinal hemorrhage constituted the sole initial manifestation. Notably, this occurred in the absence of typical constitutional symptoms such as abdominal pain, weight loss, or anorexia—a presentation exceedingly rare among reported cases (4). In the limited literature, gastric LMS presenting as acute upper gastrointestinal hemorrhage typically occurs in middle-aged to elderly patients, often mimicking GISTs endoscopically. For instance, Hasnaoui et al. reported a 63-year-old female presenting with hematemesis, melena, and life-threatening anemia (Hb 38 g/L) (5). Similarly, Gubatan and Shah described a gastric LMS unmasked by bleeding from a PEG tube in a 50-year-old male (9). Compared to these previously reported cases, our patient is older (78 years old) and presented with multiple complex cardiovascular and cerebrovascular comorbidities, which significantly increased the clinical challenge of perioperative hemodynamic management. Furthermore, unlike some larger reported hemorrhagic LMS tumors that presented as massive exophytic or ulcerated fungating masses, our patient’s tumor endoscopically appeared as a relatively small, seemingly manageable 1.8 cm submucosal lesion, which masked its true infiltrative extent and higher malignancy potential. This atypical presentation is attributable to the intraluminal protrusion of the tumor, which resulted in mucosal erosion and fissure-like ulceration, culminating in acute massive hemorrhage. This case highlights the need to consider rare gastric mesenchymal malignancies in elderly patients with unexplained acute upper gastrointestinal hemorrhage, even when classic malignant symptoms are absent. Timely endoscopic evaluation combined with contrast-enhanced cross-sectional imaging is essential to avoid diagnostic delay.

Definitive diagnosis of gastric LMS requires histopathological examination and a comprehensive immunohistochemical panel to distinguish it from other gastric mesenchymal neoplasms (1). In the current post-GIST era—where the vast majority of gastric mesenchymal tumors are classified as GISTs—the risk of misdiagnosing an exceptionally rare entity such as LMS is considerable. A recent systematic review of 41 post-GIST era cases confirmed that gastric leiomyosarcoma is vastly outnumbered by GIST and consistently exhibits an immunophenotype positive for smooth muscle markers (α-SMA, desmin) and negative for KIT and DOG1. Histologically, it is characterized by intersecting fascicles of spindle cells with marked atypia, elevated mitotic activity (13/10 HPF) including atypical mitoses, and an infiltrative growth pattern. The immunohistochemical hallmark is positivity for myogenic markers—diffuse SMA and SMMS-1, with focal desmin expression—confirming smooth muscle differentiation (4). Critically, the tumor is consistently negative for CD117 and DOG1, thereby excluding GIST, the most common gastric mesenchymal neoplasm. In the present case, the combined CD117/DOG1-negative immunophenotype, together with the diffuse and strong positivity for SMA and SMMS-1, provided unequivocal confirmation of smooth muscle differentiation and effectively excluded GIST at the immunohistochemical level. Given this definitive immunoprofile, the added diagnostic value of routine KIT/PDGFRA mutation analysis is limited, and such molecular testing is not universally mandated in standard clinical practice for cases with a definitive immunohistochemical diagnosis. Additional entities were excluded based on the morphology, immunomarkers, and molecular testing: gastric schwannoma, inflammatory myofibroblastic tumor, and benign leiomyoma. Pathologic diagnosis represents the integration of morphologic, immunohistochemical and molecular characteristics and is a key element of clinical decision making (10). The immunoprofile of the present case unequivocally established the diagnosis of LMS in accordance with current WHO criteria (1), exemplifying the standard workflow for precise pathological diagnosis of ultra-rare gastric mesenchymal malignancies in the post-GIST era.

Notably, significant discrepancies between preoperative/intraoperative assessments and final histopathology were observed in this case, involving two critical parameters: tumor size and the Ki-67 proliferation index. Preoperative gastroscopy, contrast-enhanced CT, and laparoscopic inspection each demonstrated only a focal protrusion, whereas final pathology revealed an actual tumor size of 5 × 3 × 1.5 cm. Concurrently, the Ki-67 index escalated from 25% in the superficial biopsy to 50% in the resected specimen. These underestimations are attributable to the intramural infiltrative growth pattern of gastric LMS within the muscularis propria, with limited focal intraluminal and extraluminal protrusions.

Endoscopic ultrasound was initially considered in the present case to better delineate the depth of mural invasion but was not performed. In future cases, endoscopic ultrasound, which can accurately identify the layer of origin and delineate the extent of intramural infiltration, or magnetic resonance imaging, with its superior soft-tissue contrast, may help reduce the preoperative underestimation of tumor extent and better inform surgical planning.

This growth pattern imposes two key limitations on preoperative evaluation. First, the discordance between endoscopic and laparoscopic findings arises from their opposing perspectives—mucosal versus serosal—neither of which captures the full depth of intramural infiltration. Second, superficial endoscopic biopsy sampled only the mucosal aspect of the intraluminal protrusion and cannot access the deeper tumor bulk. Coupled with the marked intratumoral morphological and proliferative heterogeneity of gastric LMS, this sampling limitation results in underestimation of mitotic activity and overall malignant potential (8). This phenomenon is not unique to gastric LMS; a recent large-scale retrospective study of 796 early gastric cancers reported an overall discrepancy rate of 25.6% between preoperative assessment and final pathological criteria, with tumor location in the upper stomach and undifferentiated histology as the primary discrepancy predictors (11). This heterogeneity was strikingly demonstrated in the present case by the Ki-67 escalation from 25% in the superficial biopsy to 50% in the resected specimen—a two-fold increase that illustrates the transition from the sampled mucosal aspect to the true proliferative core of the tumor. Such discordance underscores why histopathological assessment of the completely resected specimen remains the cornerstone of accurate grading and risk stratification. Thus, definitive diagnosis, tumor staging, and risk stratification must rely on complete histopathological examination of the en bloc resected specimen.

For localized gastric LMS, radical en bloc surgical resection with negative (R0) margins is the cornerstone of curative-intent treatment, in accordance with current ESMO and NCCN guidelines (12, 13). For tumors confined to the gastric wall without serosal invasion or distant metastasis—as in the present case—laparoscopic wedge resection represents a safe and feasible minimally invasive approach to achieve this goal. This minimally invasive approach may confer particular advantages in elderly patients with multiple comorbidities, including reduced postoperative pain and shorter hospitalization. Intraoperatively, careful exploration of the peritoneal cavity and liver—a common site of distant metastasis—is essential to exclude occult dissemination. The extent of resection should be tailored to achieve R0 margins while preserving gastric function where feasible.Routine lymph node dissection is not recommended, as lymph node metastasis in soft tissue sarcomas (including LMS) is rare (<1%), except for certain high-risk histologic subtypes (13). In the present case, the patient underwent successful laparoscopic wedge resection with R0 margins and an uneventful postoperative recovery, further supporting the safety and feasibility of this approach in carefully selected elderly patients.

Given the extreme rarity of gastric LMS, no standard adjuvant therapy regimen has been established for completely resected disease, and its role has not been clearly defined (12, 13). Adjuvant chemotherapy may be considered for selected high-risk patients, whereas the benefit of adjuvant radiotherapy in this setting remains unproven. In the present case, given the patient’s advanced age and multiple comorbidities, the potential toxicities of adjuvant therapy were carefully weighed against its uncertain benefits. Following thorough MDT discussion and shared decision-making with the patient and her family, a strategy of close surveillance was adopted in lieu of adjuvant radiotherapy or chemotherapy. The patient was discharged with a structured follow-up plan including periodic gastroscopy and chest and abdominopelvic contrast-enhanced CT.

Prognostic determinants for gastric LMS include tumor diameter (>5 cm), surgical margin status, and Ki-67 proliferation index, with R0 resection constituting the most important curative and favorable prognostic factor (14). Notably, the maximum tumor diameter in this case reached 5 cm, combined with a markedly elevated Ki-67 index of 50%—a value at the upper end of the reported range (15%–70%) (8)—both features which place the patient at high risk for recurrence. This risk profile was materially underestimated preoperatively, as the intramural component of the tumor was not fully appreciated. SEER analyses have reported 5-year cancer-specific survival rates of 66.5% following surgical resection, whereas non-surgical patients had a 0% survival rate (15). In addition to survival outcomes, large cohort studies on soft tissue sarcomas have demonstrated that the risk of distant recurrence is highest within the first two years following surgical resection, with the lungs and liver representing the predominant sites of metastasis. This recurrence pattern is consistent with the high-risk profile observed in our patient, who harbored a 5 cm tumor with an elevated Ki-67 index of 50%, and further supports the necessity of intensive early surveillance with cross-sectional imaging of the chest, abdomen, and pelvis. However, even after R0 resection, recurrence is common: one cohort of gastrointestinal LMS patients demonstrated a median recurrence-free survival of only 13 months, with 50% developing recurrent disease, predominantly as distant metastases (16). Given these high-risk features, a structured surveillance protocol was established after thorough discussion with the patient and her family, integrating guideline recommendations with the patient’s individual circumstances. It was agreed to follow current NCCN and ESMO guidelines, with surveillance imaging every 3–6 months for the first 2–3 years, then every 6 months to complete a total of 5 years, and annually thereafter (12, 13), supplemented by periodic gastroscopy to monitor for local recurrence.

Several limitations of this report should be acknowledged. First, although definitive exclusion of wild-type GIST would ideally require mutational analysis of KIT and PDGFRA, the immunohistochemical profile of the present case—characterized by dual negativity for CD117 and DOG1, coupled with strong and diffuse positivity for SMA and SMMS-1—satisfies the diagnostic criteria for LMS according to the 2020 WHO Classification of Soft Tissue and Bone Tumours. Given this definitive immunophenotypic confirmation, molecular testing was not pursued as part of the routine clinical workup. Second, the follow-up period is short (approximately one month), precluding meaningful assessment of long-term oncologic outcomes. An extended follow-up report is planned to be documented in a future update, contingent upon the patient’s continued adherence to the surveillance protocol and consent for data use. Finally, as a single case report, the generalizability of findings is inherently limited. Nevertheless, this case underscores a critical diagnostic pitfall: both superficial endoscopic biopsy and preoperative imaging may substantially underestimate the true infiltrative extent and proliferative activity of gastric LMS, highlighting the necessity of definitive histopathological evaluation of the completely resected specimen for accurate diagnosis and risk stratification.

## Conclusion

This case demonstrates that acute upper gastrointestinal hemorrhage can be the sole initial manifestation of primary gastric LMS, even in the absence of typical constitutional symptoms. The seemingly unremarkable endoscopic and preoperative imaging findings in this case masked a tumor with a considerably larger infiltrative extent and a markedly higher proliferative activity, representing a critical diagnostic pitfall. Accurate diagnosis hinges upon rigorous immunohistochemical exclusion of GIST. Multidisciplinary collaboration is essential to optimize perioperative management and postoperative surveillance in elderly patients with significant comorbidities.

## Data Availability

The original contributions presented in the study are included in the article/supplementary material. Further inquiries can be directed to the corresponding author.
